# An Efficient Targeted Drug Delivery through Apotransferrin Loaded Nanoparticles

**DOI:** 10.1371/journal.pone.0007240

**Published:** 2009-10-02

**Authors:** Athuluri Divakar Sai Krishna, Raj Kumar Mandraju, Golla Kishore, Anand Kumar Kondapi

**Affiliations:** 1 Department of Biochemistry, University of Hyderabad, Hyderabad, India; 2 Department of Biotechnology, School of Life Sciences, University of Hyderabad, Hyderabad, India; 3 Centre for Nanotechnology, University of Hyderabad, Hyderabad, India; University of Helsinki, Finland

## Abstract

**Background:**

Cancerous state is a highly stimulated environment of metabolically active cells. The cells under these conditions over express selective receptors for assimilation of factors essential for growth and transformation. Such receptors would serve as potential targets for the specific ligand mediated transport of pharmaceutically active molecules. The present study demonstrates the specificity and efficacy of protein nanoparticle of apotransferrin for targeted delivery of doxorubicin.

**Methodology/Principal Findings:**

Apotransferrin nanoparticles were developed by sol-oil chemistry. A comparative analysis of efficiency of drug delivery in conjugated and non-conjugated forms of doxorubicin to apotransferrin nanoparticle is presented. The spherical shaped apotransferrin nanoparticles (nano) have diameters of 25–50 ηm, which increase to 60–80 ηm upon direct loading of drug (direct-nano), and showed further increase in dimension (75–95 ηm) in conjugated nanoparticles (conj-nano). The competitive experiments with the transferrin receptor specific antibody showed the entry of both conj-nano and direct-nano into the cells through transferrin receptor mediated endocytosis. Results of various studies conducted clearly establish the superiority of the direct-nano over conj-nano viz. (a) localization studies showed complete release of drug very early, even as early as 30 min after treatment, with the drug localizing in the target organelle (nucleus) (b) pharmacokinetic studies showed enhanced drug concentrations, in circulation with sustainable half-life (c) the studies also demonstrated efficient drug delivery, and an enhanced inhibition of proliferation in cancer cells. Tissue distribution analysis showed intravenous administration of direct nano lead to higher drug localization in liver, and blood as compared to relatively lesser localization in heart, kidney and spleen. Experiments using rat cancer model confirmed the efficacy of the formulation in regression of hepatocellular carcinoma with negligible toxicity to kidney and liver.

**Conclusions:**

The present study thus demonstrates that the direct-nano is highly efficacious in delivery of drug in a target specific manner with lower toxicity to heart, liver and kidney.

## Introduction

Current preferred therapeutics focused on effective approaches involving radiation therapy, laser therapy, chemotherapy etc. Although surgical intervention alone or in combination with chemotherapy is a preferred treatment of solid tumors, chemotherapy alone is an easy and viable option, but its use is limited due to 1) non-specific cellular localization of drug, 2) inefficient drug release, 3) reduced effective drug concentration due to low solubility and low stability of drugs [1]–[3]. Such limitations were addressed through the development of various targeted delivery systems involving the conjugation of drug to various biomaterials such as biodegradable polymers made up of polyethylenimine [4], PLGA [5], modified liposomes [6]–[9], peptides and proteins [10], [11]. Recent developments include introduction of nanoparticles consisting of poly (ethyl amine)-b-poly(n-isopropyl acetylated derivative), nanoparticles of Fe_3_O_4_ [12], [13], polymeric dendrimers [14], [15], poly hydrogen acylated nanoparticles, core-shell co-polymer nanoparticles [16]–[18], DOTA–NHS-ester [19], PEG-PEL nanoparticles [20]. Though these materials are biocompatible, they lack target-specificity besides their high energy-demanding for degradation of biomaterial in cells and tissues. In order to enhance target specificity, many drug-loaded materials are conjugated or coated by iron binding protein transferrin/apotransferrin for recognition and facilitating binding to cancer cells [21]–[24]. Transferrin receptors are abundantly expressed in active and rapidly proliferating cells and thus targeting such proteins enables preferential localization of drugs in those cells. Cancer cells and tissues over expressing transferrin receptors has enhanced localization of drug [25], thus widening the scope of drug action on various types of cancers. The limitation in this approach of the conjugation of drug to the polymers/metal/proteins/carbohydrates in the drug delivery systems, is that the cleavage of conjugated functional group is a rate limiting step [26] and it is inefficient in drug release. The reaction conditions required for cleavage of the conjugates may work against the stability of drug [27], thus reducing the effective concentration of drug.

Apotransferrin is a stable and abundantly expressed protein that remains in circulation in blood of human beings and animals. Apotransferrin, upon binding to ferric ion, forms transferrin, which binds to transferrin receptor on the cell surface and enters the cell by receptor-mediated endocytosis [28]. The internalized transferrin is fused with endosome that followed by the release of iron into the cells. After release of iron, apotransferrin is recycled back to the cellular surface, which again participates in the transport of iron in another cycle [29]. Transferrin is a well-studied ligand for the tumor targeting [22], [30] and gene delivery [31].

The present study examines and evaluates the efficacy of apotransferrin as a sole drug carrier and compares its drug delivery efficacy in respect of the two forms - conjugated (conj-nano) and direct drug loaded nanoparticles (direct-nano). The results clearly bring out the significant advantage of direct drug loaded nanoparticles of apotransferrin in accomplishing an affective and complete drug delivery into the cells.

## Results

### Polymorphic properties of the protein nanoparticles

Nanoparticles were prepared as described in the *methods*. In this study, *nano* refer to apotransferrin nanoparticles; *direct-nano* refers to the apotransferrin nanoparticles containing doxorubicin without involving conjugation of the drug. The drug is encapsulated into the particles during preparation of nanoparticles; *conj-nano* refers to the nanoparticles comprised of apotransferrin and doxorubicin conjugates; *apo* refers to soluble apotransferrin; *direct-sol* refers to soluble mixture of apotransferrin and doxorubicin; conj-sol refers to soluble conjugates of apotransferrin and doxorubicin.

**Figure 1:**
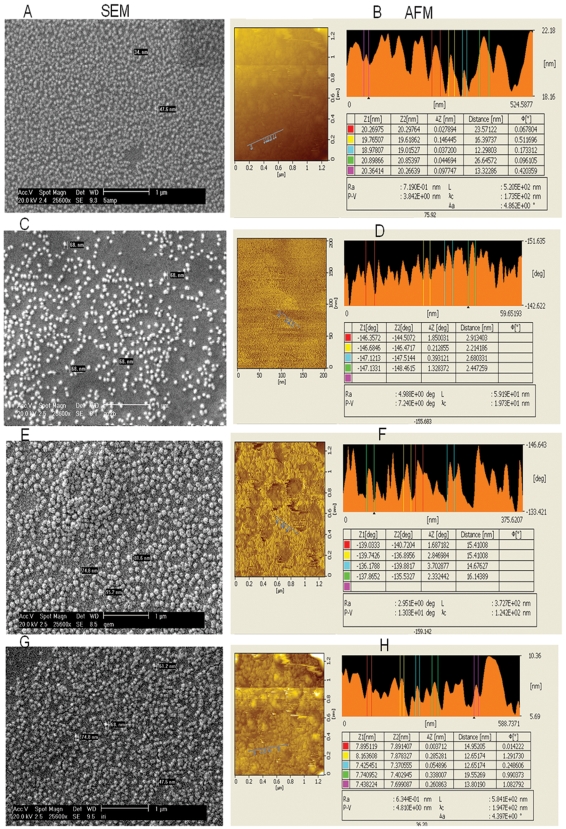
Microscopic Analysis of Nanoparticles: Apotransferrin nanoparticles were prepared as described in methods. The particles were analyzed by SEM, and AFM. Panel A depicts SEM image of nano. SEM image of direct-doxo were given in panel C. Panel E show the SEM image of conj-nano. Panel G show the SEM image of BSA nano. Panel B, D, F, H illustrates surface morphology of nanoparticles using AFM. Several independent experiments were carried out. The quality of nanoparticles used in each experiment reported in manuscript was confirmed by SEM analysis.

Analysis of nanoparticles by electron microscopy (SEM) scanning shows that these nano exhibit a uniform spherical shapes, dimension with diameters ranging from 20–40 ηm (Figure 1A). Further analysis of *nano* using atomic force microscopy (AFM) shows clear particle to particle separations in surface projections, with diameters ranging from 13 to 30 ηm (Figure 1B). Interestingly, while spherical shape of the particles remained intact in direct-nano with diameter of 60–80 ηm on SEM (Figure 1C), the particles exhibit significant surface projections of 1–3 ηm diameter (on AFM) (Figure 1D), suggesting clear polymorphic changes of the direct-nano in the presence of doxorubicin. The particle diameter (on SEM) increased to 75–95 ηm in conj-nano (Figure 1E) with correspondingly increased surface projections of 15 ηm (Figure 1F). In the case of nanoparticles made up of the albumin alone, particles were of 60–75 ηm (Figure 1G). Analysis on AFM show 12 to 19 ηm surface projections (Figure 1H) with distinctly different polymorphic features compared to those observed for *nano* (Figure 1A). These results point out that the nanoparticles are below 100 ηm diameter, and they exhibit characteristic polymorphic features, which vary with form of the nanoparticles viz., nano, direct-nano, conj-nano, BSA nano.

### Characterization of nanoparticles

The experimental results clearly indicate that protein concentration below 5 mg is not adequate for nano particle formation, while concentrations above 30 mg protein are found to result in formation of large sized particles of dimensions 200–400 ηm. It was also found that during sonication, the amplitude below 2 µm is not adequate for formation of particles. Also, the centrifugation speed is important for appropriate precipitation of particles. The particles could not be separated from oil phase at 81 x g. At 324–1296 x g pellet was formed loosely, while at very high speeds of 3968 x g and above very tight pellet was formed, which was difficult to disperse. Finally, 2025–2915 x g was found to be an optimum speed at which the particles could be collected and dispersed.

**Figure 2:**
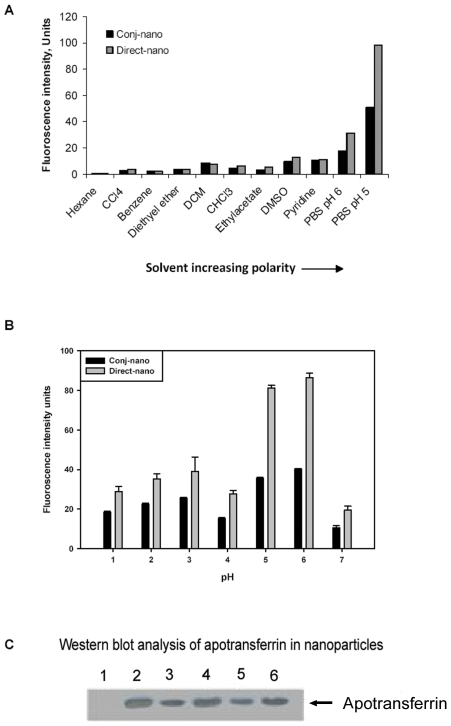
Physicochemical characterization of nanoparticles. Panel A. Surface property of nanoparticles: Sensitivity of conj-nano and direct-nano (200 µg protein equivalent) in solvents of increasing polarity (X-axis) was monitored by incubating in the presence of indicated solvent for 30 min at room temperature and doxorubicin released was measured by fluorimetry (at λ_Ex_ 470 nm λ_Em_595 nm) was plotted on Y axis. Panel B. pH dependent release of doxorubicin from nanoparticles: Direct-nano (200 µg protein equivalent) was incubated in the presence of buffers at indicated pH (X-axis) for 30 min and the amount of doxorubicin released was measured by fluorimetry (at λ_Ex_ 470 nm λ_Em_595 nm) for 30 min at room temperature. Data is represented as an average of three determinations with standard deviation. Panel C: Analysis for Apotransferrin protein in the Nanoparticles: Nanoparticles were denatured and separated on 10% SDS PAGE and western blot analysis was performed as described in methods. (1) BSA (–ve control) (2) Apo-sol (+ve Control) (3) 50 µg direct-nano (4) 100 µg direct-nano (5) 50 µg conj-nano (6) 100 µg conj-nano).

The physico-chemical properties of the particles were analyzed by treating the particles in the presence of solvents of increasing polarity and estimating the extent of doxorubicin released. If one assuming that the particles are protected by oil, the particles should be sensitive and collapse in the presence of low polar organic solvents leading to release of significant amounts of doxorubicin. The results of fluorimetry presented in Figure 2A show that the particles are indeed found to be intact in the presence of solvents having polarity as low as zero (hexane) and olive oil miscible solvent pyridine [32], and no significant amount of doxorubicin is released from particles suggesting that the particles are not protected by any oil film. Analysis of dried nanoparticles by solid IR showed no signs of olive oil in the particles (Figure S1). To further investigate, whether the particles are made of proteins, the particles were incubated in the presence of decreasing pH for 6 hours. The results of these studies clearly demonstrated that the particles are indeed made of protein alone. Since transferrin binding to receptor is a pH dependent, the ability of pH dependent release of drug from nanoparticles was studied. The results presented in figure 2B show that the particles formed both by direct-nano and conj-nano were sensitive below pH 6, maximum amount of drug release at pH 5 suggesting that a pH dependent release of drug can take place from the particles. Furthermore, the particles were immunoreactive to mouse anti-human transferrin (Figure 2C) confirming that the particles are indeed comprised of apotransferrin and the immunological epitopes of apotransferrin and are found intact for their molecular activity.

The process of protein drug nanoparticles preparation initially involves initially adsorption of the drug into the protein in the presence of oil phase leading to aggregation of drug-protein complexes. The complexes get disaggregated by sonication. The disaggregates are cooled in liquid nitrogen or on dry ice to induce their precipitation. Soluble mixtures of drug and protein, upon addition to oil phase, induce adsorption of drug to the protein followed by phase changes that lead to formation of solidified drug-protein particles. The drug may thus be loaded into cavities formed by the protein complexes through adsorption. The efficiency of the drug loading though this mode may be seen from the concentration of drug encapsulated in protein nanoparticles which is found to be as much as 500 µg in 1 mg of apotransferrin protein (50% loading). The dimension of nanoparticles loaded with drug directly is 60–80 ηm (Figure 1E), while conjugated nanoparticles show dimension of 80–100 ηm (Figure 1G). This methodology has been successfully employed in the present study to prepare apotransferrin nanoparticles of a wide range of cancer drugs, paclitaxel, irinotecan, carboplatin, oxaliplatin, etoposide etc (data not shown).

Furthermore, surface display analysis of apotransferrin nanoparticles (Figure 1B, D, F, H), shows significant surface projections indicating that the epitopic and structural projections of protein on the surface of the nanoparticles may be retained. This is also further confirmed by the intact immuno-reactivity of protein in nanoparticles with mouse-anti-human apotransferrin monoclonal antibody, as observed through western blotting (Figure 2C).

### Mechanism of apotransferrin nanoparticles mediated drug delivery

**Figure 3:**
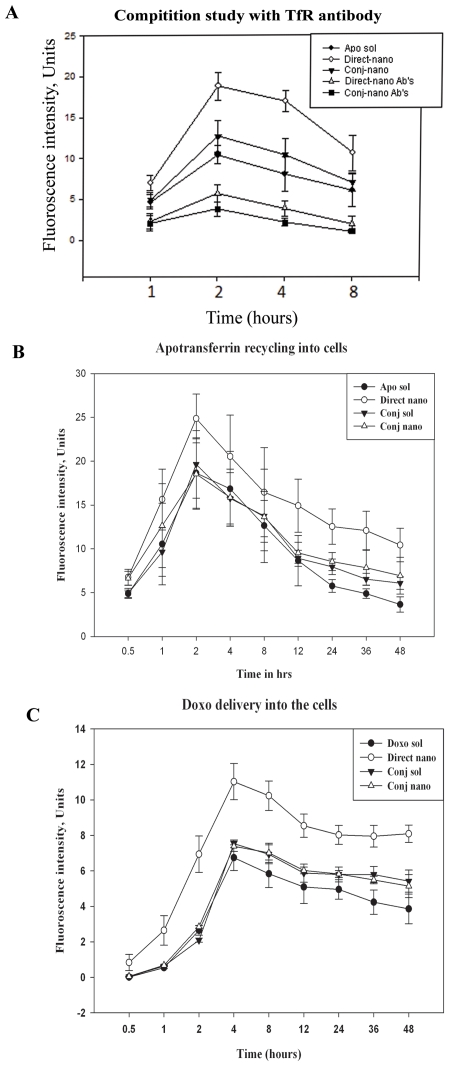
Mechanism of apotransferrin nanoparticle mediated drug delivery. Panel A. Specificity of nanopraticle interaction with transferrin receptor: SupT1 cells were incubated with indicated FITC-conjugated transferrin nanoparticles in presence or absence of mouse anti-human transferrin receptor MAb. After indicated time intervals, cells were lysed and centrifuged at 11,661 x g for 20 min at 4°C. Supernatants were collected and FITC-apotransferrin was quantified at 490/520 ηm (Ex/Em). Each data point is a mean of 3 independent determinations, the mean and standard deviation is shown with error graph. Panels B & C. Quantitative cellular localization of apotransferrin and doxorubicin: 100 µg apo-nano were added to Sup T1 cells (1 million) and incubated at different time points as indicated in figure. Cells were harvested and washed thrice with PBS and lysed in lysis buffer. Lysate was centrifuged at 11,661 x g for 20 min and supernatant was used for the quantification of FITC-apotransferrin and doxorubicin. Panel B: apotransferrin recycling in cells. Panel C: Doxorubicin loading in cells. Each data point is a mean of 3 independent determinations, the mean and standard deviation is shown with error graph.

The efficiency of delivery of doxorubicin into cells in both cases i.e., conj-nano and direct-nano, was monitored using FITC-conjugated nanoparticles. Different forms of nanoparticles were added to Sup-T1 cells and incubated for indicated time points. The amount of FITC-conjugated apotransferrin was monitored by fluorimetry (λ Ex 495 ηm and Em 521 ηm). The results presented in Figure 3A, show that in the case of the direct-nano treated cells, the doxorubicin present is at higher concentrations in the cells in a time dependent manner compared to conj-nano, suggesting an efficient drug entry through apotransferrin in direct-nano as compared to that observed in conj-nano. Furthermore, nanoparticles mediated drug entry is found to be more efficient compared to that of soluble apotransferrin (Figure 3A).

The specificity of apotransferrin nanoparticles in cellular interaction and entry was assessed by competition of nanoparticles binding in both the presence and absence of mouse anti-human transferrin receptor monoclonal antibody (1 µg/0.5 million cells). The results show that transferrin receptor specific antibody significantly inhibited the endocytosis of both direct-nano and conj-nano (Figure 3A). This suggests that the drug delivery by apotransferrin nanoparticles is mediated specifically through transferrin receptor mediated endocytosis.

We have also conducted experiments wherein doxorubicin loaded nanoparticles (both direct-nano & conj-nano) were added to Sup-T1 cells, to examine if the transferrin nanoparticles follow the same natural iron transport pathway. The amount of apotransferrin localized in the cells was monitored by quantification of FITC fluorescence (Ex: 495 ηm, Em: 521 ηm) using fluorimetry. Same cells were also monitored for doxorubicin (Ex: 470 ηm, Em: 595 ηm). The results of this study shown in (Figure 3B and C), show that direct-nano has its highest apotranferrin entry at 2 hrs after the addition of nanoparticles (Figure 3B), while the drug release maximum at 4 hrs after addition, followed by more than 50% secretion of protein by the cells within 48 hrs (Figure 3B). On the other hand, the apo-sol, doxo-sol and conj-nano showed lesser protein re-cycling and low drug release rate suggesting that conjugated form follows the localization mechanism similar to that of soluble apotransferrin.

**Figure 4:**
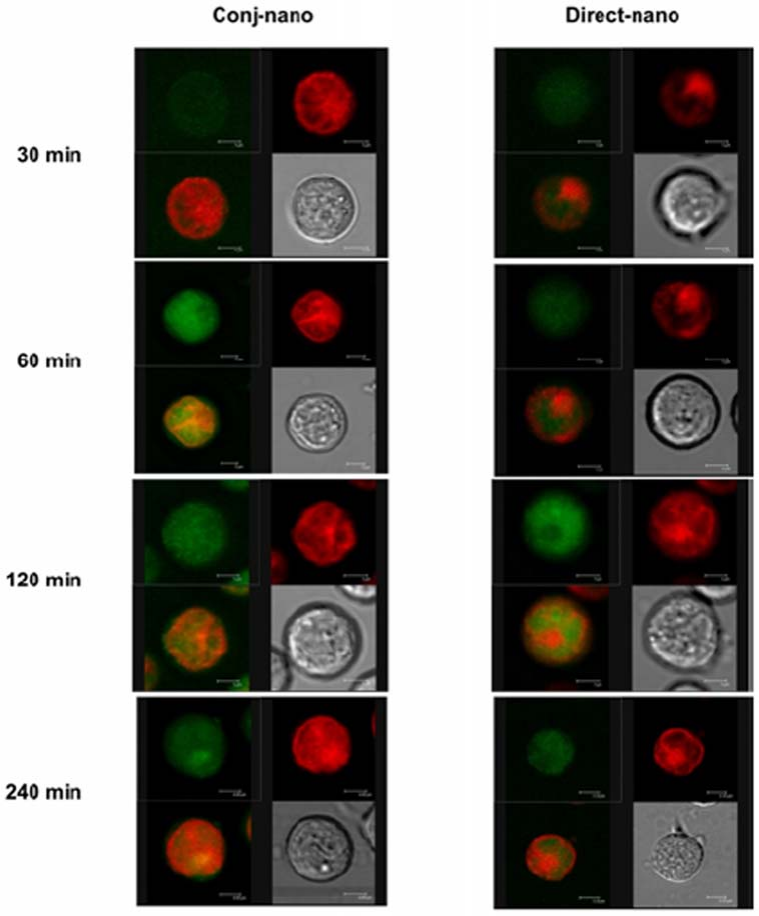
Localization of apotransferrin and doxorubicin in Sup-T1 cells. 100 µg apo-nano containing of protein-drug (conjugated as well as direct) were added to the cells (1 million) and incubated at different time points as indicated in figure. Cells were harvested at 30 min, 1 hour, 2 hours and 4 hours, then washed thrice with PBS and re-suspended in PBS. Cells were viewed in Leica confocal microscope, green fluorescence is due to the FITC-conjugated Apotranferrin and red fluorescence is due to intrinsic fluorescence of doxorubicin.

The recycling of apotransferrin observed in the targeting of doxorubicin in cancer cells was further examined and confirmed by analysis of the localization of doxorubicin and FITC-conjugated apotransferrin in nanoparticles in Sup-T1 cells by confocal microscopy. Incubation of nanoparticles for 30 min in the case of conj-nano showed the presence of doxorubicin in cytosol, possibly associated with endosomes, which released drug at 2 hours post incubation. This suggests that the drug-protein conjugates follow a mechanism similar to that of iron release through endosomes (Figure 4). In contrast, doxorubicin in direct-nano, was found localized in the nucleus (Figure 4), and was found to be stable throughout the 4 hours period of post incubation, pointing out that direct-nano can efficiently and completely localize drug in cells.

### Antiproliferative activity of apotransferrin nanoparticles

**Figure 5:**
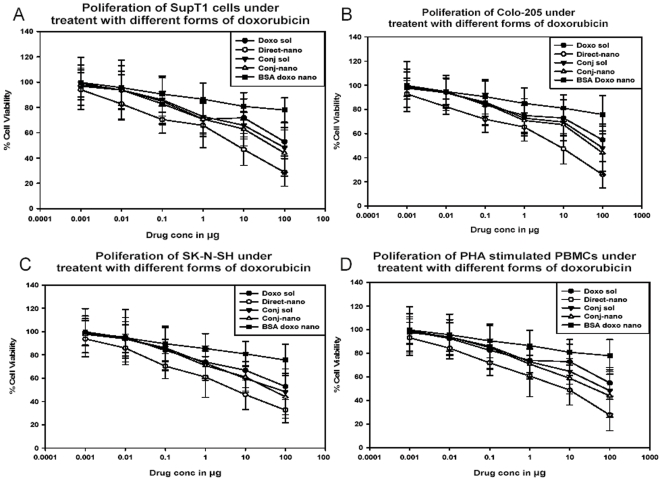
Antiproliferative activity of apotransferrin nanoparticles mediated drug Delivery. SUP-T1, Colo 205, SK-N-SH and PBMC's were incubated for 16 hours in the presence of increasing concentrations of indicated test sample. The quantity of viable cells was estimated by MTT assay. The MTT O.D value for control in absence of any test sample was taken as 100% viable. The viability of cells in each test sample was computed with reference to this value. Each data point is a mean of 3 independent determinations, the mean and standard deviation is shown with error graph.

Doxorubicin apotransferrin loaded nanoparticle forms (conjugated and nonconjugated) were tested for bioactivity in terms of anti-proliferative activity of doxorubicin against three different cancer cell lines SupT1, Colo-205 and SK-N-SH and PHA stimulated PBMC's, using MTT assay. The results (Figure 5) show that apotransferrin directly loaded nanoparticles (nonconjugated) have significantly inhibited proliferation of cells in the case of all the four types,, thus suggesting that this delivery method is free from cell type limitations for drug delivery. BSA doxo-nano exhibits relatively low activity compared to direct-nano with statistically significant differences between BSA-nano and Direct-nano at 10 µg (p<.005) and 100 µg (p<.002) of doxorubicin from Holm-Sidak pair-wise multiple comparison procedures, suggesting the importance of receptor mediated endocytosis in the case of apotransferrin.

In summary, the results of the study show a clear advantage of direct-nano in terms of rapid and complete localization of drug in the target organelle, nucleus, thus conferring potent anti-proliferative activity to the drug. This provides thus providing an efficient delivery of the drug in the case of all the four cell types examined and facilitates an effective inhibition of proliferation.

### Pharmacokinetic studies of drug release kinetics with the nanoparticles

**Figure 6:**
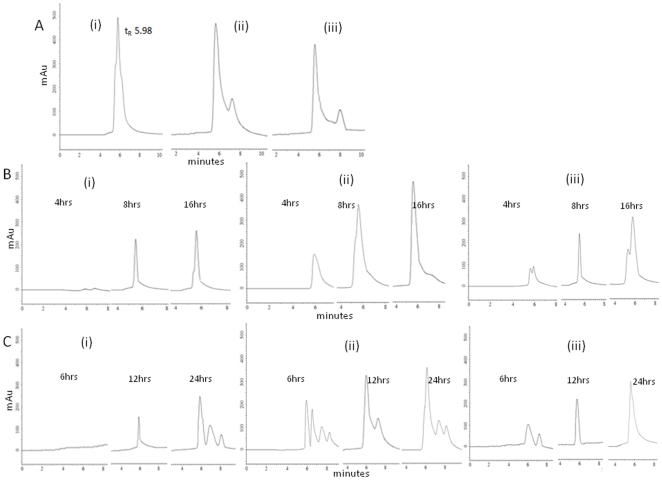
Pharmacokinetics analysis of nanoparticle based delivery of doxorubicin in rats. Rats were administered through intra-peritoneal route with direct-nano and conj-nano. Blood and liver were collected at indicated time points and processed as mentioned in the methods. Panel A: HPLC analysis of doxorubicin i) 100 ηg pure doxorubicin in methanol, ii) 100 ηg doxorubicin suspended in rat blood and extracted and subjected to HPLC, iii) 50 ng doxorubicin suspended in rat blood and extracted and subjected to HPLC. Panel B: HPLC analysis of indicted forms of doxorubicin extracted from rat blood indicated time after administration: i) soluble doxorubicin sol, ii) direct-nano, iii) conj-nano. Panel C: HPLC analysis of indicted forms of doxorubicin from liver after indicated *intra peritoneal* administration. Liver extract was prepared and doxorubicin was quantified through HPLC. i) soluble doxorubicin, ii) direct-nano, iii) conj-nano.

Nanoparticles and drug conjugates viz., direct-nano, Conj-nano, direct-sol, were administered to rats through intra peritoneal route. Drug localization in blood (Figure 6B) as well as liver (Figure 6C) was monitored. The drug in blood and in liver was extracted and estimated by HPLC analysis at 4 hrs, 8 hrs and 16 hrs post administration. Results of the HPLC analysis of pure doxorubicin and the doxorubicin isolated from blood, shown in Figure 6A clearly bring out the efficiency of the method.

Direct-nano administration did show significant drug localization in blood right from the time points 4 hrs post-administration onwards. On the other hand, the conj-nano and soluble forms showed only very low concentrations of doxorubicin at 4 hrs post administration. Further, in direct-nano administered rats, higher concentrations were detected in circulation in blood at 8 hours of post-administrations that remained stable for 16 hours. In contrast, in the case of direct-sol and conj-nano administration negligible concentrations were present at 4 hours, with levels of concentrations of doxorubicin detected becoming significant only after 8 hours of post administration that reaches maximum at 16 hours of post-administration. This suggests the doxorubicin in direct-nano is rapidly and completely released in circulation and remained stable, while the release of drug in the case of conj-nano and direct-sol is relatively slow and incomplete. Similar results have been obtained in the case of localization of drug in liver. These results clearly demonstrate that the intra peritoneal administration of direct-nano formulation of drug in rat is efficient in release of drug in circulation compared to other forms.

### Tissue localization of doxorubicin, when direct-nano and conj-nano were administered through *intravenous* route in rats

**Figure 7:**
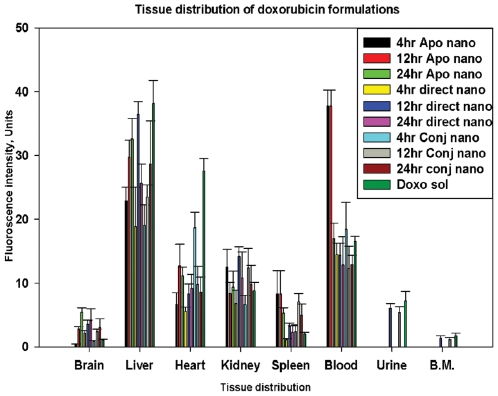
Localization of protein and doxorubicin through intravenous administration. Rats were *intravenously* administered RITC-labeled apotransferrin nanoparticles, direct-nano, conj-nano. Organs as indicated were surgically collected and RITC-apotransferrin and doxorubicin were extracted from tissue homogenate as per the procedure given in “experimental section”. Amount of RITC was estimated by fluorimetry at 573 nm (λ_em_), while amount of doxorubicin was estimated at 585 nm (λ_em_). Each data point is an average of 3 independent experiments with standard deviation is shown as error.

The localization of the doxorubicin, when administered through *intravenous* route was assessed by the administration of direct-nano and conj-nano followed by surgical removal of the organs viz. brain, liver, heart kidney, spleen and bone marrow. The doxorubicin was extracted from the tissue as per methods and estimated by fluorimetry. The results presented in Figure 7 show that the levels of doxorubicine were higher in heart, when soluble doxorubicin is administered compared that with direct-nano. Further, the doxorubicin shown to be localized higher levels in liver in direct-nano treated rat compared to conj-nano and doxo alone. Following liver, the drug localization was significant in kidney and in circulation in blood. Lowest drug concentrations have been detected in heart, brain and spleen thus reducing the risk of direct-nano other non-target organs. Since administration of doxorubicin alone is known to exhibit cardio toxicity, the current method of delivery using direct-nano reduces the localization of drug in the heart and thus provides an advantage of reducing the rise of the doxorubicin related-cardio toxicity.

### Efficacy of apotransferrin drug nanoparticles in regression of ascetic liver carcinoma in rats

Ascetic liver hepatocellular carcinoma is induced in rat by administration of 0.2 million of ZH5 cells through intra peritoneal route. In this model, the rat develops a full-blown cancer by day 4 and survives only for 7 days. The nanoparticle formulation of drug is administered as it has been shown from the pharmacokinetic studies, the drug is significantly released both circulation and liver through this mode (Figure 6). Even the intravenous administration of nanoformulation indeed showed significant levels of drug localization in liver (Figure 7).

Treatment of hepatocellular carcinoma injected rats, using the direct-nano delivery method, showed very significant regression in cancer with survivability of 150 days for 4 rats in a group of 6 rats.

The statistical significance of cancer regression in groups was analyzed by ANOVA analysis and Tukey's Honestly significant difference (HSD) test. The results presented in Table 1 showed that differences of means among the groups are significant with probability of above 95%.

**Table 1:** Efficacy of nanoparticle delivery system in regression of hepatocellular carcinoma in rats.

Parameters Average (SD)	Untreated	Direct-nano*	Direct- sol*	Doxo sol*	BSA nano*	Saline/healthy rats*
**Serum Lactate Dehydrogenase (IU/L)**	206.48 (4.01)	51.68 (1.03)	89.89 (0.84)	90.19 (1.10)	182.04 (3.34)	51 (0.73)
**Serum Alkaline Phosphatase (IU/L)**	252.01 (1.36)	60.49 (0.81)	71.33 (0.52)	123.97 (4.09)	184.86 (3.05)	57.02 (0.04)
**Serum Creatinine (mg/dl)**	0.23 (0.01)	0.19 (0.02)	0.20±0.01	0.20 (0.01)	0.20±0.006	0.21 (0.01)
**Serum Urea (mg/dl)**	26.05 (0.86)	4.66 (0.04)	3.63 (0.007)	4.58 (0.007)	19.21 (0.53)	4.62 (0.13)
**Survival time (days)**	7 (1)	No sign of death	16 (1)	16 (1)	8 (1)	No sign of death

SD: Standard deviation; Doxo: Doxorubicin. Each group has 6 animals. (*) 10 days treatment. Differences between the groups were analyzed by ANOVA. Tukey's HSD test was carried out for pair wise comparison of mean values of each group. Data is presented in supporting data.

A group of 6 rats were induced cancer and indicated agent (Table 1) was administered intra peritoneal from day-1 of post-induction for a period of 6-days and rats were sacrificed at day-6 and the relevant parameters were estimated in liver, kidney and urine. The results presented in Table-1 clearly bring out that direct-nano treated rats showed a significant regression of tumor. Further, the results of toxicity to liver and kidney in the case of direct-nano treated rats are similar to those of untreated placebo, while treatment with soluble doxorubicin and direct-sol show low survival rates compared to direct-nano (Table 1). These results suggest that soluble forms of doxorubicin in the presence and absence of apotransferrin has no significant advantage. BSA-nano did not show significant regression of cancer as per Tukey's HSD pair-wise comparison analysis between BSA nano and direct-nano treated rats, thus suggesting the importance of receptor recognition in transferrin-mediated endocytosis of direct-nano in specific. In general, target specificity would determine the site of drug delivery through protein nanoparticles *in vivo*.

Since serum alkaline phosphate plays a significant role in tissue and cellular damage in liver, it serves as an important indicator for assessing the cancer-induced liver toxicity in hepatocellular carcinoma progression. The results of analysis of alkaline phosphatase in nanoparticle treated rat amounts to 60.49±0.81 IU/L, which comes close to that for healthy ones, 57.02±0.04 IU/L (Tukey HSD difference 3.47±10.43 between healthy and direct-nano nanoparticle treated). The treatment through direct-nano nanoparticles can thus have the advantage of inhibiting cell and tissue damage in liver. The enzyme level is four fold higher in untreated rat (252.01±1.36 IU/L with Tukey HSD difference is 191.51±10.43).

The direct-nano nanoparticles treated rats have also shown significant decrease in the levels of the prognostic hepatoma marker serum, lactate dehydrogenase (LDH). While it was 206±4 IU in untreated rats (Tukey HSD difference is 154.8±10.76), it has decreased to, 51.68±1 IU/L, a value close to that of healthy rats (Table 1). To monitor, metabolic disorder, if any, caused upon induction of hepatocellular carcinoma, serum creatinine and urea were estimated in rats. The creatinine in treated rats remained the same as that for healthy as well as direct-nano drug treated rat (Tukey HSD difference less than 0.04). The serum urea in untreated rats increased significantly by 5 fold (Tukey HSD difference 21.43±1.92), while it remained low in healthy and direct-drug nanoparticle treated rats (Table-1) suggesting that treatment did not induce any toxicity to kidney. The observed decrease of efficacy of doxorubicin in BSA nanoparticles could be due to their inability in cellular binding and release of doxorubicin (Table 1). This indicates that receptor recognition and binding of apotransferrin nanoparticles play an important role in drug release. The results thus show that direct-drug nanoparticles can significantly and efficiently regress cancer in rat hepatocellular carcinoma and this is further confirmed by their longer survival rate (Table 1) and immunohistochemistry (Figure S2).

## Discussion

Cells express two types of Transferrin receptors,TfR1 and TFR2, TfR1 is expressed on all nucleated cells including hepatocytes, but with differences in level of expression [33]. Altered iron metabolism is shown to favor TfR1 and is over expressed in Hepatocellular carcinoma (HCC) in rats [34] and in tumors of human HCC patients [35]–[37]. Since HCC is diagnostically frequently detected at advanced stage and short time is available for intervention, the current research efforts are focused mainly on the development of carriers to allow a rapid and efficient delivery of drug, which is an immediate need for treatment of HCC. Emerging cancer therapies involve use of conjugated drug delivery systems with polymeric materials, proteins, carbohydrates and other biodegradable materials [38]. The present study aimed at developing a new approach for targeted delivery of drugs to cancer in general and HCC in particular, investigates the efficacy of doxorubicin direct-nano in treatment of hepatocellular carcinoma based on a rat cancer model through a localized administration by *intra peritoneal* route.

The results showed that the apotransferrin nanoparticles of dimensions 30–50 ηm, can efficiently and quantitatively load the drug and when loaded they assume a dimension of 60–80 ηm in the case of doxorubicin.

Further, the particle size is inferred to be dependent on the globular nature of the protein as BSA nano particles (globular) showed higher dimensions compared to direct-nano made of apotransferrin. The surface properties of the nanoparticles depend on the form of the protein species present, that is, whether it is in conjugated or non-conjugated form. The doxorubicin in both direct-nano and conj-nano modes is shown localized into cells through transferrin receptor mediated endocytosis, with a relatively higher concentration of drug delivery by the direct- nano. This in turn is succeeded by secretion of the apotransferrin protein from the cell facilitating removal of the delivery vehicle apotransferrin from the cell, thus reducing burden to the cell from delivery vehicle. The direct-nano mode is found to be highly efficient in delivery of drug through *intra-peritoneal* route, as significant amount of drug was observed in circulation at 4 hrs of post administration, reaching a maximum at 8 hrs and remained stable at that level for 16 hrs compared to conj-nano and doxo-sol, which showed low concentrations of drug release. The anticancer activity of direct-nano was also found to be significantly high on different cell lines tested. Also, the intravenous administration is found to be efficient in delivery of drug to liver suggesting that this may be considered as a possible route of application of this formulation in human studies as well for treatment of HCC or any liver cancer in future.

Doxorubicin is known to cause cardio toxicity [39], but tissue distribution studies through *intravenous* administration of direct-nano and conj-nano, showed that the highest localization of the doxorubicin occurred in liver and kidney compared to other organs especially lower levels are found in the heart. Further, the level of doxorubicin in heart decreased with time in case of direct-nano, in contrast to an increase with time in the case of soluble doxorubicin. Due to such lower drug localization in heart, the direct- nano delivery method reduces the risk of doxorubicin related cardio toxicity.

The mechanisms of transportation of drug into tumors from the bloodstream are yet to be fully understood [40]. The nanoparticle uptake of the organs like liver and spleen is mainly based on their surface characteristics [41]. Particles with more hydrophobic surfaces are preferentially taken up by the liver, followed by the spleen and lungs [42]. Hydrophilic nanoparticles (35 ηm diameter), such as those prepared from poly(vinyl pyrrolidone), show less than 1% uptake by the spleen and liver and show as much as 5–10% of particles still circulating in the bloodstream even at 8 hrs after injection. This study shows that significant concentrations of drug is localized in liver, blood and kidney (Fig. 6 &7), while protecting the HCC bearing animals from cancer-induced toxicity to both liver and kidney (Table 1). However, nanoparticles composed of 50% PNVP and 50% n-isopropyl acrylamide (45 or 126 ηm diameter) also showed preferential uptake by the liver [43], [44]. It has been shown that coatings of hydrophilic polymers can create a cloud of chains at the particle surface which will repel plasma proteins, may be involved in specific cellular localization [45]. Other routes include formulations of the particles from branched or block copolymers with hydrophilic and hydrophobic domains [46]. The heterogeneity of blood flow in non-necrotic regions of tumors is highly relevant and any addition of even slower and unpredictable blood flow in necrotic and semi-necrotic regions only compounds the complexity of the challenging task of drug delivery to cancerous tissues. In this context, the apotransferrin drug delivery system presented here will form a new class of drug delivery systems originated from a natural cellular transport pathway without involving any conjugation to the drug. Further, this is a new low-toxic approach for fast and complete drug delivery, as compared to some of the conjugated drug delivery systems. This technology can be applied to any protein ligand that has a specific receptor on the target cell. Furthermore, the use of allogenic protein from the same patient will prevent the rare immuno-rejection of the delivery vehicle.

## Materials and Methods

### Materials

Apotransferrin was purified from human blood following the method of Cohn *et al.* [47]. Doxorubicin was a pharmaceutical preparation of Biochem Pharmaceutical Industries, Pune, India. SUP-T1 cells were from NIH-AIDS reference and reagents program, USA. SK-N-SH, COLO-205 cells were from NCCS Pune, India. PBMC's were isolated as described in [48]; All other reagents and biochemicals were of analytical and molecular biological grade.

### Animals

All animal experiments were conducted as per approval of the Institutional Animal Ethics Committee (IAEC) of the University of Hyderabad. Wister rats (Age: 6–8months, Weight: 0.15–0.175 kg) were maintained in the university animal house. Apotransferrin nanoparticles loaded drugs were injected through intra peritoneal and intravenous routes. After indicated time points, blood, liver, kidney, heart, spleen, brain and other organs were collected as per the approval of the IAEC and drug was extracted by silver nitrate and methanol method.

### Nanoparticle (Direct nano) Preparation

For preparation of nanoparticles, procedure described in Kondapi *et al.*, [49] was adopted. 25 mg of apotransferrin dissolved in 100 µl of phosphate buffer saline (PBS) or dimethyl sulphoxide (DMSO). The solution of apotransferrin (100 µl) was slowly mixed with a 100 mM of doxorubicin hydrochloride (3.46 mg in 100 µl DMSO) and the mixture was incubated on ice for 5 min. The mixture of apotransferrin and the drug was slowly added to 30 ml of olive oil at 4°C with continuous dispersion by gentle vortexing. The sample was sonicated at 4°C using an MSE sonicator probe (PG43301, MSE Instruments, UK) or equivalent with a 30 sec period pulse, having an amplitude of 5 µm. This sonication step was repeated 15 times with a gap of 1 minute between successive steps. The resulting mixture was immediately frozen in liquid nitrogen for 10 min. and was then transferred to ice and incubated for 4 hours. The particles formed were pelleted by centrifugation at 2915 x g for 10 min at 4°C and the pellet was extensively washed with diethyl ether and dispersed in phosphate buffered saline (PBS). The particles were then estimated for protein using Biuret method and the nanoparticles were expressed in terms of protein concentration equivalents. The possibility of protein or doxorubicin entrapment in the form of emulsion along with the nanoparticles was assessed through the study of solubilization of the final pellet for water or DMSO soluble doxorubicin by fluorimetry and apotransferrin by Biuret method. No significant soluble forms of doxorubicin or apotransferrin were found thus ruling out the possibility of any emulsion formation during the particle preparation. Nanoparticles in pellet form were stable. When dispersed in PBS they were stable for 1 week at room temperature and for 2 months at 4°C and 6 months at −80°C.

### Optimization of the nanoparticle preparation

Direct nanoparticles were prepared as described in “Direct-nano” under varying conditions of sonication, centrifugation, and protein concentration and aqueous to oil ratio. The particles were washed extensively with ether and dispersed in PBS followed by the analysis of particles using SEM.

### BSA nanoparticles

BSA nanoparticles were prepared as described in “direct nano” preparation (as explained above) using 25 mg of albumin from bovine serum (Sigma chemical co.) in place of apotransferrin.

### Fluorescence/rodamine isothiocyanate (FITC/RITC) Conjugation of apotransferrin

Apotransferrin was conjugated with FITC/RITC as described in product manual of Sigma Chemical Co.. FITC/RITC-conjugated apotransferrin was used directly for the preparation of nanoparticles.

### Doxorubicin conjugation with apotransferrin

Doxorubicin was conjugated with apotransferrin by coupling with EDC (1-ethyl-3-[3-dimethylaminopropyl] carbodiimide hydrochloride). 10 mg of purified apotransferrin in ultrapure water was mixed with 3.46 mg (100 mM) of doxorubicin and 10 mg of EDC. The contents were mixed thoroughly and reaction was carried out for 2 hrs at room temperature. Non-conjugated drug was removed by dialysis (membrane with 12 KDa cut–off) against PBS. Buffer was changed 4 times with a 6 hr time interval. The conjugated protein, after dialysis, was used for experiments and for preparation of conjugated nanoparticles.

### Characterization of Nanoparticles

Structure and morphology of the nanoparticles were investigated using Scanning electron microscope (Philips FEI-XL 30 ESEM, USA - operated at 20 kV), Transmission electron microscope (JOEL JEM 1011, USA - operated at 100 kV), and Atomic force microscope (SPA-400, USA); manufacturers' instructions were followed for sample preparation, data collection, and analysis of particles as describe below.

### Transmission electron microscopy (TEM) [50]

Unstained samples of a apotransferrin drug loaded/unloaded nanoparticles were prepared for electron microscopy measurements/observations, by air-drying small drops of a sample solution onto carbon-coated copper electron microscopy grids, to obtain stained images of protein nanoparticles, the electron microscopy grids containing air-dried samples were incubated with a 2% (w/v) aqueous uranyl acetate solution for 10 min at room temperature and washed 3–4 times with distilled water. Protein nanoparticle images were examined using the JOEL JEM 1011 100 kV electron microscope. Electron diffraction patterns were recorded from a selected area that is well occupied with protein nanoparticles in order to obtain high diffraction intensities. Particle size distributions were made by measuring diameters for 50 protein nanoparticles.

### Scanning Electron Microscopy [51]

Nano particle samples were analyzed using a PHILIPS FEI-XL ESEM (USA), operated at 100 kv). Metal stubs were coated with double-sided adhesive tape and nanoparticles were dispersed on the sticky surface. Specimen was air dried in dust free environment at room temperature for one hour, then coated with gold in Sputter Coater. Specimens were stored in dry, dust free environment during the analysis. Images were recorded using appropriate resolution.

### Atomic force microscopy [52]

Glass piece (0.5×0.5 mm) is placed on to one side of a double stick tape and on to the other side of the tape a mica sheet is attached. The mica sheet with a cellophane tape stuck on it is pulled out to peel it and removal of layer of mica sheet was repeated till several times to obtain a smooth surface of mica sheet. A 5 µl of the sample was uniformly dispersed using a spin coater and dried in a dust free zone for 12 hours. The unit containing the sample is kept in SPA-400 and images were recorded at different resolutions and surface morphology was analyzed following manufacturer's instructions.

### Western blot analysis

50–100 µg of protein equivalent nanoparticles were denatured by heating at 65°C in the presence of SDS loading buffer and separated on 7.5% sodium dodecyl sulphate (SDS) gel and then transferred to nitrocellulose membrane. Western blotting was carried out as described in Pierce for Western Blot Chemiluminescence Reagent [53]. The blot was developed using mouse anti-human transferrin Mab (Abcam). The relative levels of protein in different lanes were compared by analyzing scanned images using the IMAGEJ (NIH) program. The analysis was repeated for a minimum of three times using independent cultures.

### Competition of the transferrin receptor antibodies with the Apotransferrin-drug Nanoparticles

One million cells were incubated in serum free media for 30 min in a 12 well plate. FITC labeled Apotransferrin-drug nanoparticles (equivalent to 50 µg of protein) were added to Sup-T1 cells in the presence as well as absence of mouse anti-human transferrin receptor antibody (Calbiochem, 2 µg/million of cells) and incubated for different intervals of time ranging from 1–8 hrs. After incubation, the cells were washed thrice with PBS and lysed in 1 ml of lysis buffer. The lysate was cleared by centrifugation at 11,661 x g for 20 min at 4°C. Fluorescent emission of the FITC (λ Ex 495 ηm and Em 521 ηm) and doxorubicin (λ Ex 480 ηm and Em 596 ηm) was measured by fluorimeter (Shimadzu FL 2000). Concurrent results of three independent observations are reported along with standard deviation.

### Nanoparticle Recycling Assay

1×10^6^ SUP-T1 cells were incubated for 30 min in serum free RPMI medium and FITC labeled apotransferrin nanoparticles were added to these cells and the incubation was continued for 30 more minutes. The cells were pelleted by centrifugation at 116 x g for 5 minutes. The pellet was extensively washed with PBS and transferred to a fresh 12 well plate in 10% serum containing media. The cells were harvested at different time points and washed thrice with PBS and lysed in lysis buffer. The lysate was cleared by centrifugation at 11,661 x g for 20 min at 4°C. Fluorescent emissions of both the FITC (λ Ex 495 ηm and Em 521 ηm) and those of doxorubicin (λ Ex 480 ηm and Em 596 ηm) were quantified using fluorimetry (Shimadzu FL 2000). Each of the above experiments was independently repeated three times to check the reproducibility; the data is presented in terms of average with standard deviation.

### Cellular localization of apotransferrin

One million SUP-T1 cells were incubated for 30 min in serum free RPMI medium and FITC labeled apotransferrin nanoparticles were added to these cells and the incubation was continued for 30 min, 1–4 hours respectively. After incubation, the cells were pelleted by centrifugation at 116 x g for 5 minutes. The pellet was washed thrice with PBS and resuspended in PBC and monitored on Leica confocal microscope.

### MTT assay

This assay was carried out using SUP-T1/Colo-205/SK-N-SH/PBMCs, as per [54].

### Pharmacokinetics and tissue distribution studies

Estimation of doxorubicin in rat blood and tissue samples was performed as described in [55] with minor modifications as described below.

#### Standard solutions of doxorubicin

Doxorubicin hydrochloride was used to prepare 1 mg/ml stock standard solution in methanol. The stock solution was stored at 4°C.

#### Mobile phase

The buffer, pH 4.0, was prepared from 10 mM ammonium hydrogen phosphate solution, to which 5 mL trimethyl amine was added. The pH of the solution was adjusted to 4.0 with orthophosphoric acid.

The mobile phase was a mixture of buffer pH 4.0/acetonitrile/methanol (60∶24∶16 [V/V/V]), sonicated for 10 min (Biologics ultrasonic cleaner, India) and filtered through 0.22 µm filter.

#### Reagents

Aqueous silver nitrate solution (30% *m/V*) was prepared by dissolving silver nitrate in purified water. 100 µL of the reagent was used as protein precipitant. Aqueous trichloroacetic acid (TCA) (10% *m/V*) was prepared by dissolving trichloroacetic acid in purified water. 100 µL of the reagent was used as protein precipitant. DNA solution (1 mg/mL) was prepared by dissolving salmon sperm DNA as model DNA (Hi-Media, India) in purified water. 100 µL of the reagent was used for the interaction studies.

#### Instrumentation

The HPLC system (Shimadzu, Japan) used in this study consists of a Waters 2690 separations module (comprising a pump, a refrigerated auto sampler with a 20 µL loop) and a Shimadzu RF-10 AXL fluorescence detector (Shimadzu Corporation, Japan). The detector was set at 480 nm and 560 nm (excitation and emission wavelengths, respectively). The detector was set at gain 3 and sensitivity 1. Chromatographic separation was performed on a 150×4.6 mm i.d. reversed-phase C18 column. Separation of analytes was performed at a flow rate of 1.3 mL/min^−1^, and at a typical back pressure of 12.67 MPa.

#### Blood samples

Standard solution of doxorubicin hydrochloride (Doxo) was added to 400 µL rat whole blood (containing EDTA) in a glass tube in respective amounts to obtain the final concentrations of 10, 40, 80, 160, 240 and 480 ng/mL of Doxo. To this mixtures 100 µL of 30% AgNO_3_ was added. The contents were vortexed for 1 min and 5 mL methanol was added. The mixture was extracted for 10 min and centrifuged at 2000 rpm for 10 min at 20°C. The supernatant was decanted into another glass tube and evaporated to dryness at 60°C under a stream of nitrogen. The dried residue was reconstituted with 200 µL methanol and centrifuged at 15000 rpm for 10 min. Clear supernatant was collected in HPLC vials and loaded onto the HPLC system.

#### Tissue Samples

Tissues extracts of liver and brain were prepared in the extraction buffer. 100 µL of 30% AgNO_3_ was added to 600 µl of tissue extract. The contents were vortexed for 1 min and 5 mL methanol was added. The mixture was extracted for 10 min and centrifuged at 2000 rpm for 10 min at 20°C. The supernatant was decanted into another glass tube and evaporated to dryness at 60°C under a stream of nitrogen. The dried residue was reconstituted with 200 µl methanol and centrifuged at 15000 rpm for 10 min. Clear supernatant was collected in HPLC vials and loaded onto the HPLC system.

### Rat Hepatocellular carcinoma model generation

A group of 6 rats was taken for each of the drug concentration and the experiments were done as per the guidelines approved by the Animal Ethics Committee, University of Hyderabad. A sample of 100 million ZH5 cells (Obtained from Centre for cellular and Molecular Biology, Hyderabad) in 0.5 ml of peritoneal fluid was taken and injected into intra peritoneal cavity to the 2 month old Wister rat (120–140 gms). It is known, that the rat develops ascetic hepatocellular carcinoma symptoms within 4 days and by 7^th^ day it will lose the activity and the animal will die during 7–8^th^ day due to multiple metastasis and hepatocellular carcinoma [56].

#### Dosage schedule

The drug was administered at a rate of one dose per day, for 10 days through intra peritoneal route using 31-gauge insulin syringe (BD biosciences).

#### Effective dose

250 µg of protein equivalent- direct-nano containing 125 µg of doxorubicin. The particles were dispersed in 0.5 ml of PBS and were administered through intra peritoneal route to the 120–140 g Wister rat.

### Sample collection and analysis

Treated as well as untreated rats were anaesthetized and sacrificed by standard cervical dislocation method, and blood samples were collected by heart puncture method and a range of biochemical parameters were estimated.

### Estimation of biochemical indicators in serum

Estimation of Urea was done by Bertholet method [57], Creatinine by picrate method [58], Alkaline phosphatase by PNPP method [59], LDH [60] by the kits supplied by Qualigens diagnostics, which are manufactured by Sigma diagnostics (India).

### Statistical analysis

The above markers were estimated in six individual rats. The significance was analyzed by Analysis of variance (ANOVA) to test whether the mean values among the groups were significantly different Tukey's honestly significant difference (HSD) test was carried out for pair wise comparison of mean values of each group. Pairwise multiple comparison procedure of Holm-Sidak was used for pair-wise analysis of results of MTT studies and tissue distribution studies using Sigma Plot v.11.0.

## Supporting Information

Figure S1 Infrared spectroscopic analysis of dried sample of nanoparticle pellet. Nanoparticles were formed as described the methods. The ether washed nanoparticle pellet was dried and FT IR spectrum was recorded. The results shown below clearly indicate the that the nanoparticles do not possess any oil film.
(3.84 MB DOC)

Figure S2 Immunochemical analysis of cancer tissue treated with doxorubicin in nanoformulation. Treated and untreated rats were anaesthetized and sacrificed by standard cervical dislocation method, and blood sample was collected by heart puncture method and liver tissue was collected and the samples were immediately washed thrice with PBS and kept in 4% Para formaldehyde solution. These samples were embedded in paraffin wax and processed for Haematoxylin/eosin staining and the specimen was photographed and analysed. The results show a significant efficacy of direct-nano compared to soluble doxorubicin and soluble mixture of doxorubicin and apotransferrin.
(0.29 MB DOC)

## References

[1] Minguez B, Tovar V, Chiang D, Villanueva A, Llovet JM (2009). Pathogenesis of hepatocellular carcinoma and molecular therapies.. Curr Opin Gastroenterol.

[2] Lopez-Tarruella S, Martin M (2009). Recent advances in systemic therapy. Advances in adjuvant systemic chemotherapy of early breast cancer.. Breast Cancer Res.

[3] Hait WN, Hambley TW (2009). Targeted cancer therapeutics.. Cancer Res 69: 1263–1267; discussion.

[4] Kircheis R, Wightman L, Schreiber A, Robitza B, Rossler V (2001). Polyethylenimine/DNA complexes shielded by transferrin target gene expression to tumors after systemic application.. Gene Ther.

[5] Xu P, Gullotti E, Tong L, Highley CB, Errabelli DR (2009). Intracellular drug delivery by poly(lactic-co-glycolic acid) nanoparticles, revisited.. Mol Pharm.

[6] Udhrain A, Skubitz KM, Northfelt DW (2007). Pegylated liposomal doxorubicin in the treatment of AIDS-related Kaposi's sarcoma.. Int J Nanomedicine.

[7] Wong HL, Bendayan R, Rauth AM, Wu XY (2006). Simultaneous delivery of doxorubicin and GG918 (Elacridar) by new polymer-lipid hybrid nanoparticles (PLN) for enhanced treatment of multidrug-resistant breast cancer.. J Control Release.

[8] Xu JP, Ji J, Chen WD, Shen JC (2005). Novel biomimetic polymersomes as polymer therapeutics for drug delivery.. J Control Release.

[9] Ying XY, Du YZ, Chen WW, Yuan H, Hu FQ (2008). Preparation and characterization of modified lipid nanoparticles for doxorubicin controlled release.. Pharmazie.

[10] Tian Z, Zhang AY, Ye L, Wang M, Feng ZG (2008). Preparation and evaluation of a linoleic-acid-modified amphiphilic polypeptide copolymer as a carrier for controlled drug release.. Biomed Mater.

[11] Anhorn MG, Wagner S, Kreuter J, Langer K, von Briesen H (2008). Specific targeting of HER2 overexpressing breast cancer cells with doxorubicin-loaded trastuzumab-modified human serum albumin nanoparticles.. Bioconjug Chem.

[12] Chen B, Sun Q, Wang X, Gao F, Dai Y (2008). Reversal in multidrug resistance by magnetic nanoparticle of Fe3O4 loaded with adriamycin and tetrandrine in K562/A02 leukemic cells.. Int J Nanomedicine.

[13] Munnier E, Cohen-Jonathan S, Linassier C, Douziech-Eyrolles L, Marchais H (2008). Novel method of doxorubicin-SPION reversible association for magnetic drug targeting.. Int J Pharm.

[14] Papagiannaros A, Dimas K, Papaioannou GT, Demetzos C (2005). Doxorubicin-PAMAM dendrimer complex attached to liposomes: cytotoxic studies against human cancer cell lines.. Int J Pharm.

[15] Kono K, Kojima C, Hayashi N, Nishisaka E, Kiura K (2008). Preparation and cytotoxic activity of poly(ethylene glycol)-modified poly(amidoamine) dendrimers bearing adriamycin.. Biomaterials.

[16] Wei JS, Zeng HB, Liu SQ, Wang XG, Tay EH (2005). Temperature- and pH-sensitive core-shell nanoparticles self-assembled from poly(n-isopropylacrylamide-co-acrylic acid-co-cholesteryl acrylate) for intracellular delivery of anticancer drugs.. Front Biosci.

[17] Yadav AK, Mishra P, Jain S, Mishra AK, Agrawal GP (2008). Preparation and characterization of HA-PEG-PCL intelligent core-corona nanoparticles for delivery of doxorubicin.. J Drug Target.

[18] Hsieh MF, Cuong NV, Chen CH, Chen YT, Yeh JM (2008). Nano-sized micelles of block copolymers of methoxy poly(ethylene glycol)-poly(epsilon-caprolactone)-graft-2-hydroxyethyl cellulose for doxorubicin delivery.. J Nanosci Nanotechnol.

[19] Mier W, Hoffend J, Kramer S, Schuhmacher J, Hull WE (2005). Conjugation of DOTA Using Isolated Phenolic Active Esters: The Labeling and Biodistribution of Albumin as Blood Pool Marker.. Bioconjugate Chemistry.

[20] Yadav AK, Mishra P, Mishra AK, Jain S, Agrawal GP (2007). Development and characterization of hyaluronic acid-anchored PLGA nanoparticulate carriers of doxorubicin.. Nanomedicine.

[21] Thorstensen K, Romslo I (1993). The transferrin receptor: its diagnostic value and its potential as therapeutic target.. Scand J Clin Lab.

[22] Qian ZM, Li H, Sun H, Ho K (2002). Targeted drug delivery via the transferrin receptor-mediated endocytosis pathway.. Pharmacol Rev.

[23] Li H, Sun H, Qian ZM (2002). The role of the transferrin-transferrin-receptor system in drug delivery and targeting.. Trends Pharmacol Sci.

[24] Vincent A, Babu S, Heckert E, Dowding J, Hirst SM (2009). Protonated Nanoparticle Surface Governing Ligand Tethering and Cellular Targeting.. ACS Nano.

[25] Gatter KC, Brown G, Trowbridge IS, Woolston RE, Mason DY (1983). Transferrin receptors in human tissues: their distribution and possible clinical relevance.. J Clin Pathol.

[26] Braslawsky GR, Kadow K, Knipe J, McGoff K, Edson M (1991). Adriamycin(hydrazone)-antibody conjugates require internalization and intracellular acid hydrolysis for antitumor activity.. Cancer Immunol Immunother.

[27] Beyer U, Roth T, Schumacher P, Maier G, Unold A (1998). Synthesis and in vitro efficacy of transferrin conjugates of the anticancer drug chlorambucil.. J Med Chem.

[28] Ali SA, Joao HC, Hammerschmid F, Eder J, Steinkasserer A (1999). Transferrin trojan horses as a rational approach for the biological delivery of therapeutic peptide domains.. J Biol Chem.

[29] Stein BS, Bensch KG, Sussman HH (1984). Complete inhibition of transferrin recycling by monensin in K562 cells.. J Biol Chem.

[30] Singh M (1999). Transferrin As A targeting ligand for liposomes and anticancer drugs.. Curr Pharm Des.

[31] Misra S, Hascall VC, De Giovanni C, Markwald RR, Ghatak S (2009). Delivery of CD44 shRNA/nanoparticles within cancer cells: perturbation of hyaluronan/CD44v6 interactions and reduction in adenoma growth in Apc Min/+ MICE.. J Biol Chem.

[32] Bills CE (1926). Fat solvents.. J Biol Chem.

[33] Enns CA, Suomalainen HA, Gebhardt JE, Schroder J, Sussman HH (1982). Human transferrin receptor: expression of the receptor is assigned to chromosome 3.. Proc Natl Acad Sci USA.

[34] Holmström P, Gåfvels M, Eriksson LC, Dzikaite V, Hultcrantz R Expression of iron regulatory genes in a rat model of hepatocellular carcinoma.. Liver Int.

[35] Tseng HH, Chang JG, Hwang YH, Yeh KT, Chen YL Expression of hepcidin and other iron-regulatory genes in human hepatocellular carcinoma and its clinical implications.. J Cancer Res Clin Oncol 2009 Apr 23. [Epub ahead of print].

[36] Yuen MF, Hou JL, Chutaputti A Asia Pacific Working Party on Prevention of Hepatocellular Carcinoma. Hepatocellular carcinoma in the Asia pacific region.. J Gastroenterol Hepatol.

[37] Aravalli RN, Steer CJ, Cressman EN Molecular mechanisms of hepatocellular carcinoma.. Hepatology.

[38] Hamad I, Moghimi SM (2008). Critical issues in site-specific targeting of solid tumours: the carrier, the tumour barriers and the bioavailable drug.. Expert Opin Drug Deliv.

[39] Plosker GL, Faulds D (1993). Epirubicin. A review of its pharmacodynamic and pharmacokinetic properties, and therapeutic use in cancer chemotherapy.. Drugs,.

[40] Jain RK (1999). Transport of molecules, particles, and cells in solid tumors.. Annu Rev Biomed Eng.

[41] Zamboni WC (2008). Concept and clinical evaluation of carrier-mediated anticancer agents.. Oncologist.

[42] Brigger I, Dubernet C, Couvreur P (2002). Nanoparticles in cancer therapy and diagnosis.. Adv Drug Deliv Rev.

[43] Gaur U, Sahoo SK, De TK, Ghosh PC, Maitra A (2000). Biodistribution of fluoresceinated dextran using novel nanoparticles evading reticuloendothelial system.. Int J Pharm.

[44] Storm G, Belliot SO, Daemen T, Lasic DD (1995). Surface modification of nanoparticles to oppose uptake by the mononuclear phagocyte system.. Advanced Drug Delivery Reviews.

[45] Romberg B, Hennink WE, Storm G (2008). Sheddable coatings for long-circulating nanoparticles.. Pharm Res.

[46] Grayson SM, Godbey WT (2008). The role of macromolecular architecture in passively targeted polymeric carriers for drug and gene delivery.. J Drug Target.

[47] Cohn EJ, Strong LE, Hughes WL, Mulford DJ, Ashworth JN (1946). Preparation and Properties of Serum and Plasma Proteins. IV. A System for the Separation into Fractions of the Protein and Lipoprotein Components of Biological Tissues and Fluids1a,b,c,d.. Journal of the American Chemical Society.

[48] Iankov ID, Blechacz B, Liu C, Schmeckpeper JD, Tarara JE (2007). Infected cell carriers: a new strategy for systemic delivery of oncolytic measles viruses in cancer virotherapy.. Mol Ther.

[49] Kondapi AK Novel nanoparticles of apotransferrin/transferring pharmaceutical composition containing them and their process for preparation (1572/CHE/2006)..

[50] Baalousha M, Motelica-Heino M, Galaup S, Le Coustumer P (2005). Supramolecular structure of humic acids by TEM with improved sample preparation and staining.. Microsc Res Tech,.

[51] Lochmann D, Weyermann J, Georgens C, Prassl R, Zimmer A (2005). Albumin-protamine oligonucleotide nanoparticles as a new antisense delivery system. Part 1: physicochemical characterization.. Eur J Pharm Biopharm.

[52] Erickson B, DiMaggio SC, Mullen DG, Kelly CV, Leroueil PR (2008). Interactions of poly(amidoamine) dendrimers with Survanta lung surfactant: the importance of lipid domains.. Langmuir.

[53] Towbin H, Staehelin T, Gordon J (1979). Electrophoretic transfer of proteins from polyacrylamide gels to nitrocellulose sheets: procedure and some applications.. Proc Natl Acad Sci U S A.

[54] Kondapi AK, Satyanarayana N, Saikrishna AD (2006). A study of the topoisomerase II activity in HIV-1 replication using the ferrocene derivatives as probes.. Arch Biochem Biophys.

[55] Reddy LH, Meda N, Murthy RR (2005). Rapid and sensitive HPLC method for the estimation of doxorubicin in dog blood–the silver nitrate artifact.. Acta Pharm.

[56] Pande G, Joshi DS, Sundaram K, Das MR (1986). Isolation and characterization of the two subpopulations of cells with different lethalities from Zajdela ascitic hepatoma.. Cancer Res.

[57] Berthelot, P.E M (1859). Berthelot's reaction mechanism,. Report Chim Appl.

[58] Bowers LD, Wong ET (1980). Kinetic Serum Creatinine Assays. II.. A Critical Evaluation and Review, Clin Chem.

[59] Bowers GN,  McCommb RB (1972). Study of optimum buffer conditions for measuring alkaline phosphatase activity in human serum.. Clin Chem.

[60] Babson AL, Babson SR (1973). Kinetic Colorimetric Measurement of Serum Lactate Dehydrogenase Activity.. Clin Chem.

