# Supplementary material for: An Efficient Targeted Drug Delivery through Apotransferrin Loaded Nanoparticles
Source: PLoS One. 2009 Oct 2;4(10):e7240. doi: 10.1371/journal.pone.0007240 (PMC2752169; doi:10.1371/journal.pone.0007240)
Supplement: Figure S1 — Infrared spectroscopic analysis of dried sample of nanoparticle pellet. Nanoparticles were formed as described the methods. The ether washed nanoparticle pellet was dried and FT IR spectrum was recorded. The results shown below clearly indicate the that the nanoparticles do not possess any oil film. (3.84 MB DOC) [file pone.0007240.s001.doc]

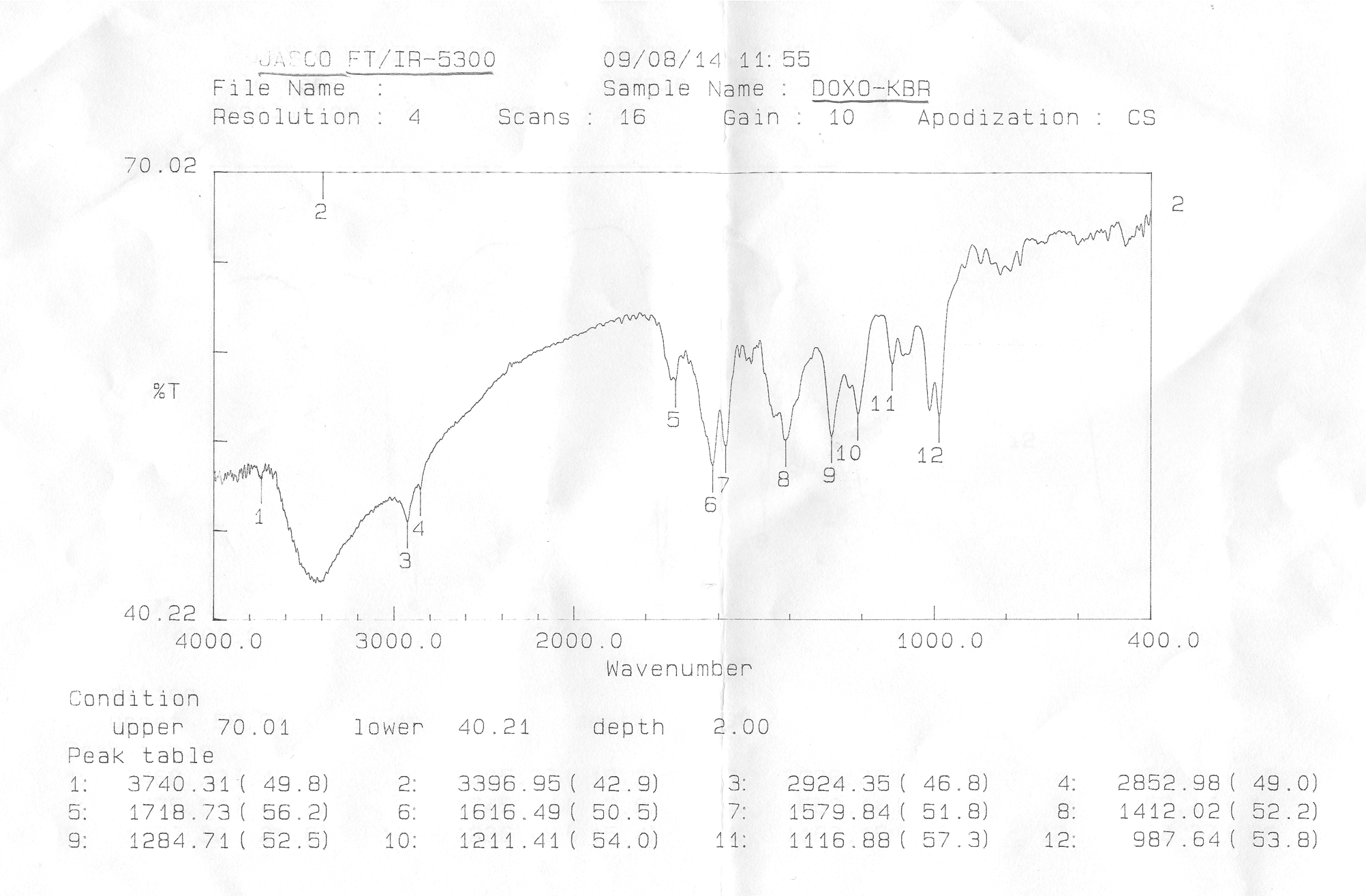


1. Doxorubicin


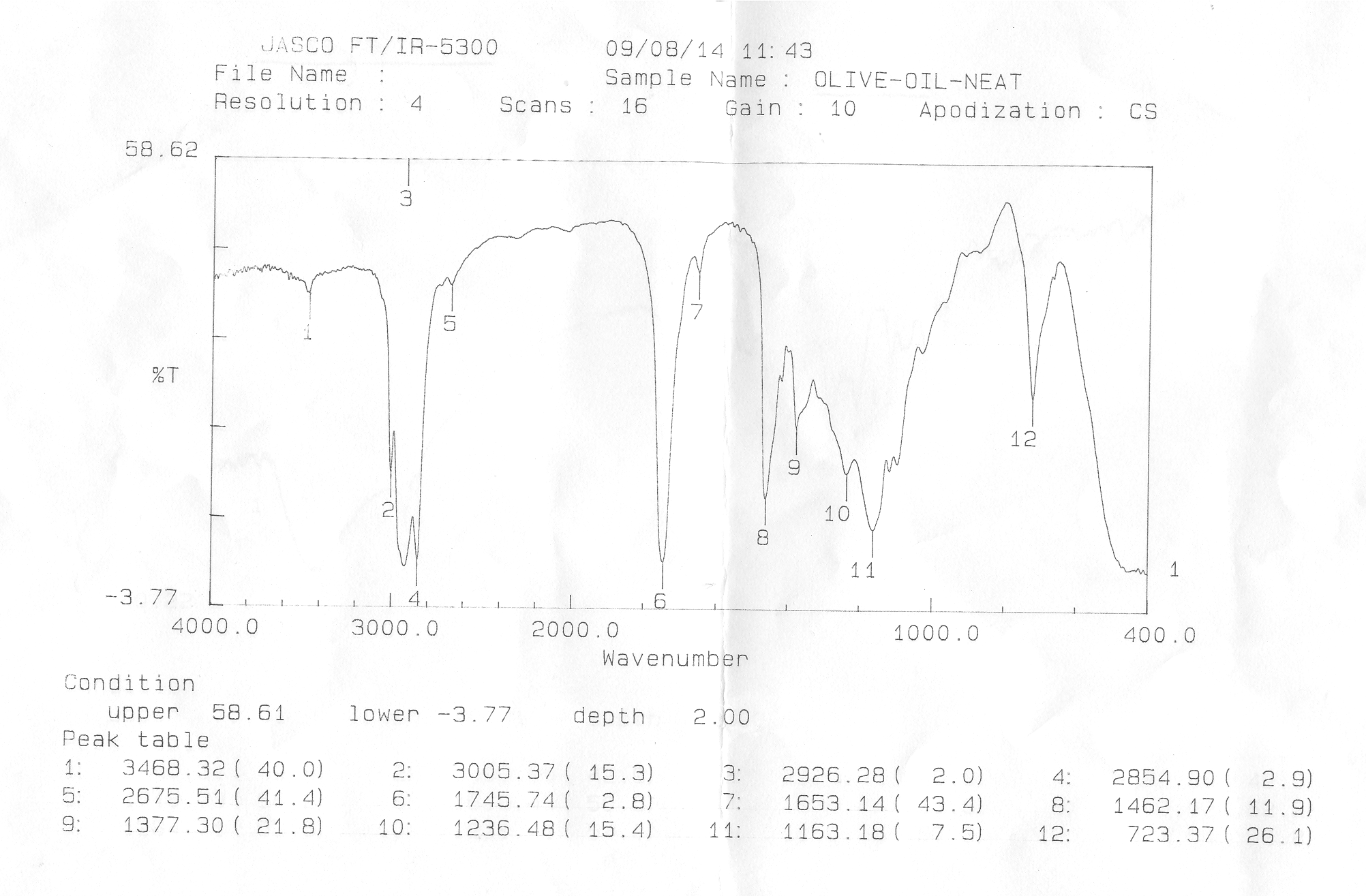


1. Olive Oil


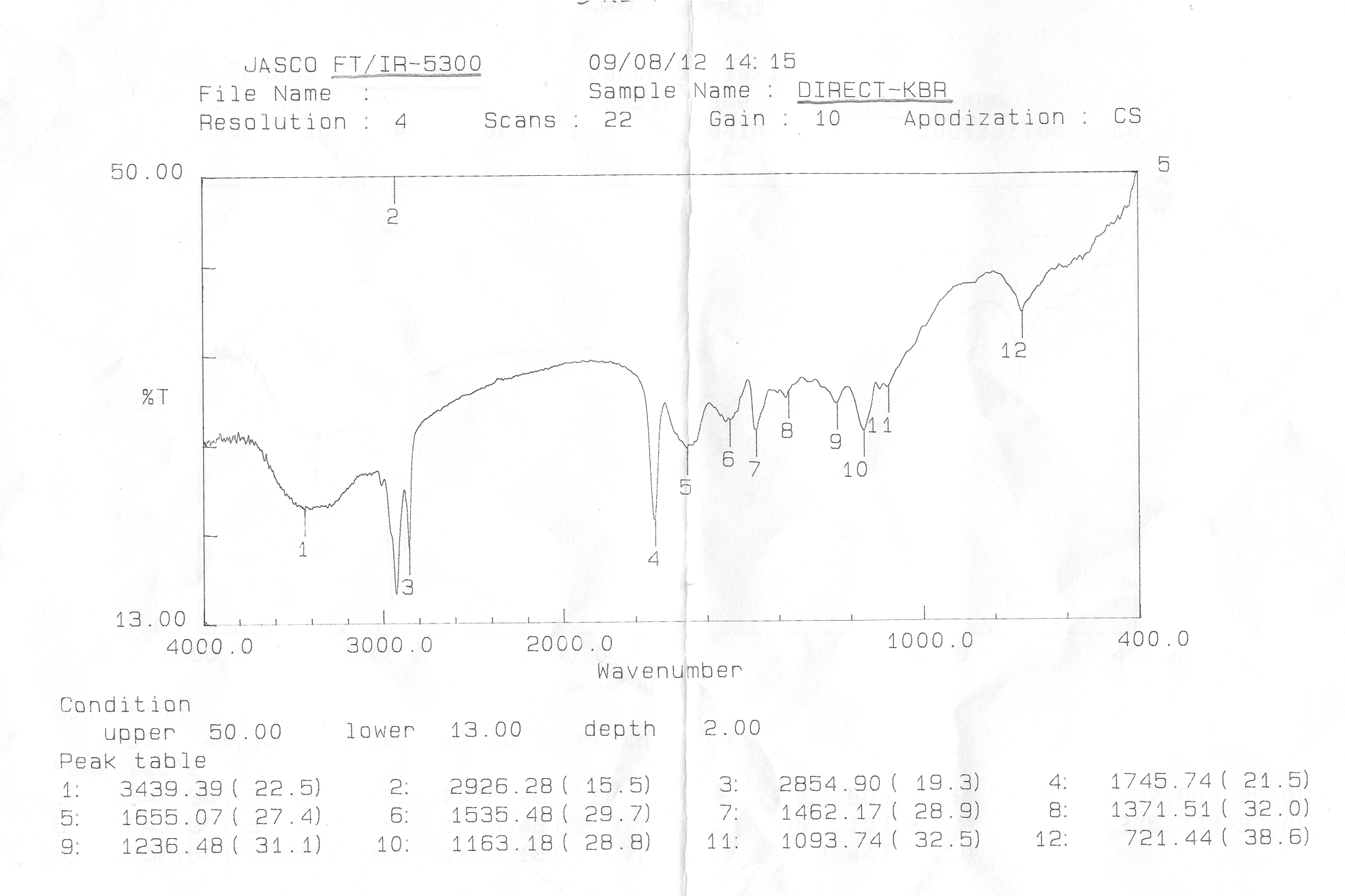


1. Direct nanoparticles


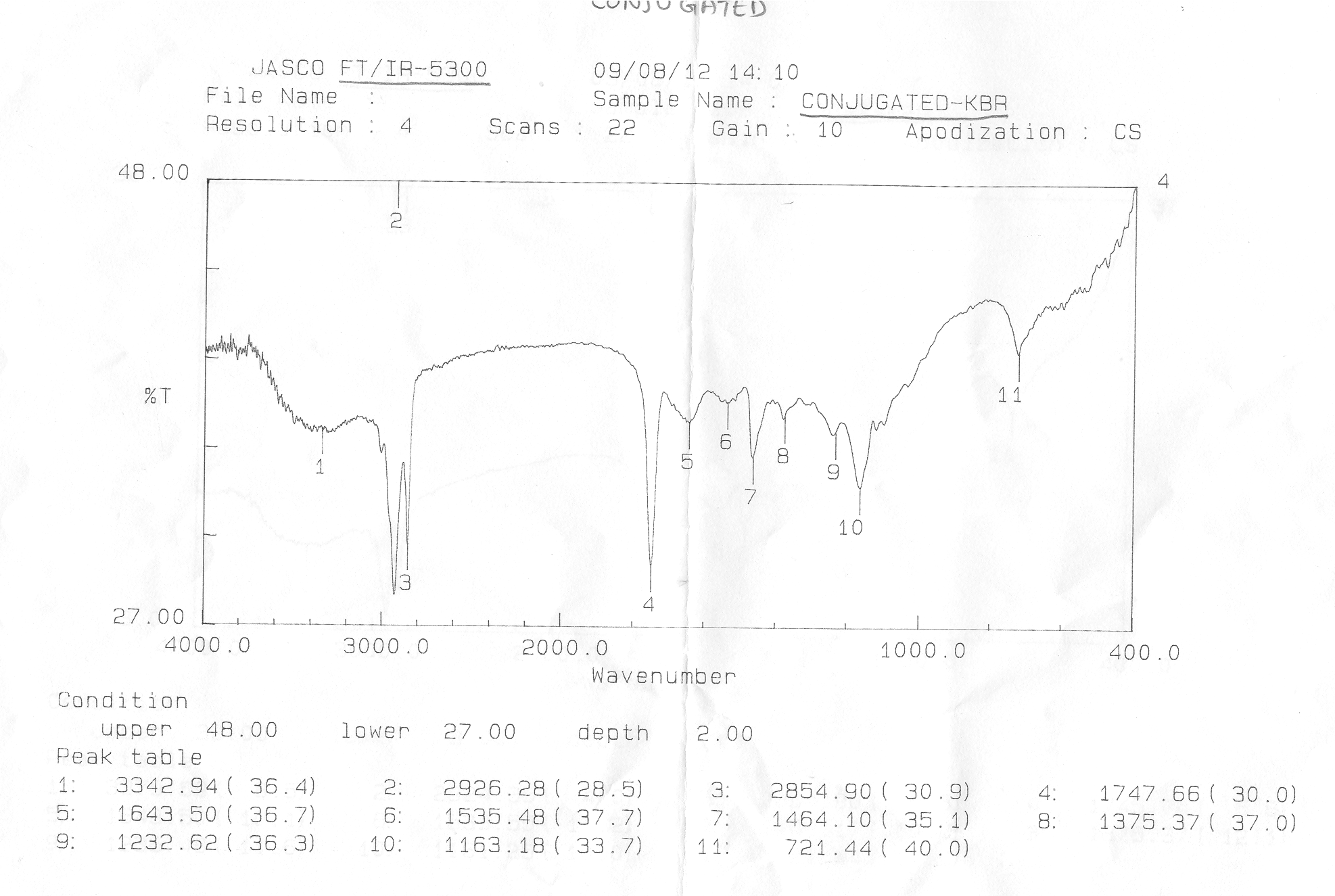


1. Conjugated nanoparticles


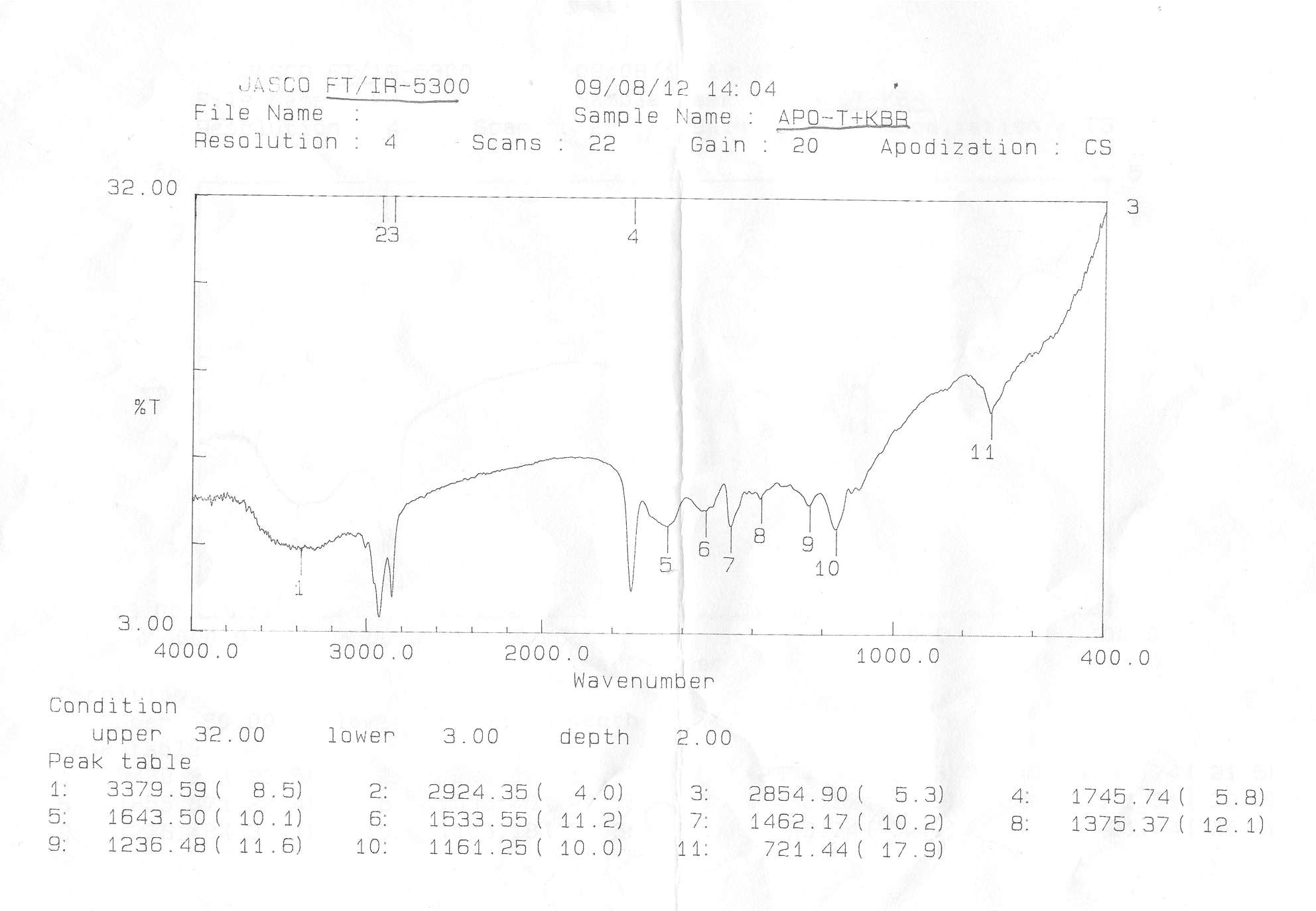


1. Apotransferrin Nanoparticles
