# Supplementary figures and images for: An Efficient Targeted Drug Delivery through Apotransferrin Loaded Nanoparticles
Source: PLoS One. 2009 Oct 2;4(10):e7240. doi: 10.1371/journal.pone.0007240 (PMC2752169; doi:10.1371/journal.pone.0007240)

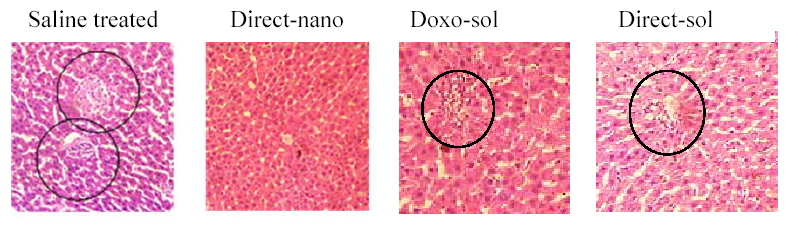

Supplement: Figure S2 — Immunochemical analysis of cancer tissue treated with doxorubicin in nanoformulation. Treated and untreated rats were anaesthetized and sacrificed by standard cervical dislocation method, and blood sample was collected by heart puncture method and liver tissue was collected and the samples were immediately washed thrice with PBS and kept in 4% Para formaldehyde solution. These samples were embedded in paraffin wax and processed for Haematoxylin/eosin staining and the specimen was photographed and analysed. The results show a significant efficacy of direct-nano compared to soluble doxorubicin and soluble mixture of doxorubicin and apotransferrin. (0.29 MB DOC) [file pone.0007240.s002.doc]
